# SNP@Evolution: a hierarchical database of positive selection on the human genome

**DOI:** 10.1186/1471-2148-9-221

**Published:** 2009-09-05

**Authors:** Feng Cheng, Wei Chen, Elliott Richards, Libin Deng, Changqing Zeng

**Affiliations:** 1 Beijing Institute of Genomics, Chinese Academy of Sciences, Beijing, PR China; 2 Graduate School of the Chinese Academy of Sciences, Beijing, PR China; 3 Department of Biology, College of Life Sciences, Brigham Young University, Provo, UT, USA; 4 Current address: Baylor College of Medicine, Houston, TX, USA; 5 Current address: Medical College of Nanchang University, Nanchang, Jiangxi, PR China

## Abstract

**Background:**

Positive selection is a driving force that has shaped the modern human. Recent developments in high throughput technologies and corresponding statistics tools have made it possible to conduct whole genome surveys at a population scale, and a variety of measurements, such as heterozygosity (HET), *F*_*ST*_, and Tajima's D, have been applied to multiple datasets to identify signals of positive selection. However, great effort has been required to combine various types of data from individual sources, and incompatibility among datasets has been a common problem. SNP@Evolution, a new database which integrates multiple datasets, will greatly assist future work in this area.

**Description:**

As part of our research scanning for evolutionary signals in HapMap Phase II and Phase III datasets, we built SNP@Evolution as a multi-aspect database focused on positive selection. Among its many features, SNP@Evolution provides computed *F*_*ST *_and HET of all HapMap SNPs, 5+ HapMap SNPs per qualified gene, and all autosome regions detected from whole genome window scanning. In an attempt to capture multiple selection signals across the genome, selection-signal enrichment strength (E_S_) values of HET, *F*_*ST*_, and *P*-values of iHS of most annotated genes have been calculated and integrated within one frame for users to search for outliers. Genes with significant E_S _or *P*-values (with thresholds of 0.95 and 0.05, respectively) have been highlighted in color. Low diversity chromosome regions have been detected by sliding a 100 kb window in a 10 kb step. To allow this information to be easily disseminated, a graphical user interface (GBrowser) was constructed with the Generic Model Organism Database toolkit.

**Conclusion:**

Available at , SNP@Evolution is a hierarchical database focused on positive selection of the human genome. Based on HapMap Phase II and III data, SNP@Evolution includes 3,619,226/1,389,498 SNPs with their computed HET and *F*_*ST*_, as well as qualified genes of 21,859/21,099 with E_S _values of HET and *F*_*ST*_. In at least one HapMap population group, window scanning for selection signals has resulted in 1,606/10,138 large low HET regions. Among Phase II and III geographical groups, 660 and 464 regions show strong differentiation.

## Background

Natural selection has played an essential role in the formation of the human genome and in the diversity of phenotypes. The identification of the functional targets of positive selection, however, has been a major challenge in understanding the evolution of human beings. Traditional investigations to localize loci that have undergone selection have focused on the coding sequences of individual genes. For example, since the finding of hemoglobin B gene under the selective pressure for malaria resistance, only a limited number of genes, including *G6PD*, *LCT*, and *ASPM*, have been functionally determined as targets of positive selection [1-3]. In recent years, the rapid development in high throughput DNA technologies, as well as in statistical analysis and bioinformatics tools, has promoted whole genome surveys in multiple aspects of genetic variation [4-6]. Since the construction of the human HapMap, many massive genome-wide projects aiming to search and scan for SNPs, indels, copy number variations (CNVs), functional DNA elements, DNA methylation sites, and expression quantitative trait loci (eQTL) have been accomplished or undertaken [7-17].

These enormous maps and datasets have made great contributions to trace our evolutionary history. Although only a handful of genome-wide measurements with limited marker density have been developed to detect selection signals, these initial studies suggest that large scale and highly detailed analyses will greatly illuminate our understanding of human evolution [18-21]. Using whole genome SNP data, selection signals may be demonstrated through the computation of classical measurements including heterozygosity (a measurement which is used to estimate the frequency of heterozygote in a population, also referred to as HET), Tajima's D (a statistical test which is used to determine whether a genetic locus is under neutral selection), and the fixation index (a measurement which is used to compare the genetic variability within and between populations, also referred to as *F*_*ST*_) [18,22,23]. Moreover, haplotype based measurements--such as extended haplotype homozygosity (EHH) and relative EHH (REHH), or more complex values including integrated haplotype homozygosity (iHH) and integrated haplotype score (iHS)--have been successfully applied to find signals of recent positive selection across the human genome [24,25]. A few databases, such as Haplotter and SNP@Ethnos, serve as public tools in population genetics [26]. Built to find ethnically related SNPs, SNP@Ethnos provides 100,736 individual variants with large ethnic differences. With the determination of ancestral or derived allele state, Haplotter is efficient in finding recent positive selections and their affected genes by the frequency of haplotypes that extend from a core SNP.

To complement these and other publicly available databases, we have built an integrated data library on human evolution ("SNP@Evolution") which offers several novel features: (1) the inclusion of E_S _(enrichment strength of selection-signals) to estimate the selection strength on a specific gene by computation of outlier ratios; (2) a sliding window scanning method that uses measures of HET and *F*_*ST *_to locate genomic segments under selection (with the resulting regional signals reflecting geographical adaptation, founder effects, and fixed or unfixed selections); (3) HapMap Phase III data from samples of 11 populations; (4) the integration with a haplotype-based dataset of iHS that simplifies the process of comparing multiple datasets by providing a simple, easy-to-read table.

One of SNP@Evolution's primary functions is to find selection signals from both chromosome regions and individual SNPs. To achieve this aim, our major strategy has involved the computation and comparison of HET, *F*_*ST*_, and iHS of all SNPs and their regional values using a sliding window method, then followed by the demonstration of outliers of each measurement as the selection signals. E_S _values of various measurements in most annotated genes are also listed as an independent dataset. Selection signals can therefore be detected in the data query or visualization interface by (1) SNP outliers, (2) low HET regions merged from adjacent window outliers, and (3) E_S _values of individual genes. With the comparison and integration of multiple datasets, SNP@Evolution provides candidate genes and regions that will assist researchers in locating positive selection signals genome-wide.

## Construction and content

### Data source

**Figure 1:**
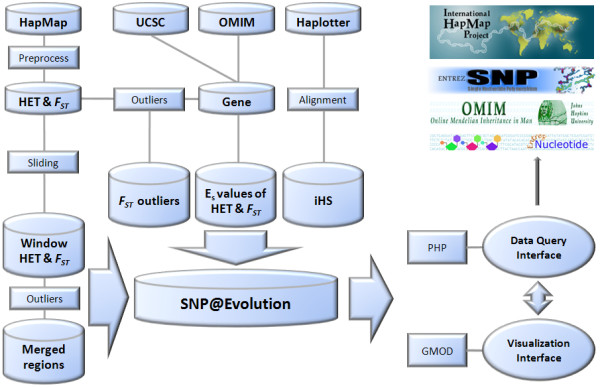
**Data collecting and processing strategy for building SNP@Evolution**. HapMap, UCSC, OMIM, and Haplotter were major sources for SNP, Gene, and iHS. After computation and processing with a sliding window and empirical distribution method, we obtained five types of outcomes that may contain evolutionary signals in the human genome including low HET or highly differentiated regions, *F*_*ST *_outliers of SNPs, E_S _values of HET and *F*_*ST *_on genes, and SNP outliers of iHS. In order to visualize these datasets, we used PHP and GMOD to build the table and figure interfaces. Links to the public online databases, including Entrez Nucleotide, dbSNP, OMIM, and HapMap, are provided together with the query results in SNP@Evolution.

The strategy of our database construction and data processing route is illustrated in Fig. 1. Genotype datasets are derived from the International HapMap Phase II and Phase III data repository (release 21# NCBI build35, release 24# NCBI build36, release 26# NCBI build36, ) [7,8]. Only data of unrelated individuals were utilized. For HapMap Phase II, samples of 60 Utah residents with ancestry from northern and western Europe (CEU), 45 Han Chinese in Beijing (CHB), 45 Japanese in Tokyo (JPT), and 60 Yoruba in Ibadan, Nigeria (YRI) were included. Considering the great genetic similarity between CHB and JPT, we pooled the data of both as one geographical group and denoted it as ASN (Asian). Phase III data came from samples of 11 populations, including 84 CHB, 85 Chinese in Metropolitan Denver, Colorado (CHD), 86 JPT, 113 CEU, 88 Toscans in Italy (TSI), 113 Yoruba in Ibadan, Nigeria (YRI), 53 African ancestry in Southwest USA (ASW), 90 Luhya in Webuye, Kenya (LWK), 143 Maasai in Kinyawa, Kenya (MKK), 88 Gujarati Indians in Houston, Texas (GIH), 50 Mexican ancestry in Los Angeles, California (MEX). According to their continental origination, samples were then divided into four geographical groups, i.e., CHB, CHD, and JPT grouped as ASN (Eastern Asian ancestry); CEU and TSI as EUR (European ancestry); YRI, ASW, LWK, and MKK as AFR (African ancestry); and GIH and MEX as AME (Native American ancestry). The iHS dataset (from HapMap phase II), including iHS of SNPs and *P*-values on iHS from 12,683 genes, was retrieved from Haplotter [25].

### Data analysis

We first computed HET and Akey's *F*_*ST *_to measure the polymorphism within each population and the differentiation among geographical groups [18]. For gene analysis, we considered a genic region to be a gene with 2 kb flanking regions. Genic regions containing no less than 5 genotyped SNPs in the dataset were chosen for subsequent analysis. From 24,011 annotated genes in NCBI build36, we identified 21,859 and 21,099 qualified genic regions in HapMap Phase II and Phase III, respectively.

To search for outliers of HET and *F*_*ST*_, the SNPs with HET values larger than average were considered as outliers directly. For *F*_*ST*_, we defined an "outlier" as a SNP whose value extends beyond the nearest quartile by a length of at least 1.5 times the inter-quartile range. With simulated data, we first sought to determine whether this method (henceforth referred to as the "empirical distribution method") is efficient and reliable to obtain selection signals without having to consider the apriori distributions. Genotype data under different selection strength and neutral selection generated by SelSim were mixed together [27], then the empirical distribution method was applied to find outliers. The results indicated that the empirical distribution method separated data under positive selection from the mixed data pool with a high sensitivity, stability, and low error rate (manuscript in preparation). (To test the empirical distribution, the positive predictive value, or PPV, can be used to describe the proportion of outliers that have been the targets of selection [28]. For instance, when the simulated proportion of selection target is 0.05 with a certain recombination environment--Rec rate = 10^-8^, the PPV of the empirical distribution ranges from 0.50 to 0.90 under the selection strength 20-200.) Therefore, we used the empirical distribution method to identify SNP outliers of *F*_*ST *_as well as the window outliers of HET (see below).

The ratios of outlier numbers of both HET and *F*_*ST *_in each genic region to the corresponding total SNPs were then calculated. Relevant enrichment value E_S _of both HET and *F*_*ST *_were determined as the percentile values on the distribution of outlier ratios for all qualified genes. Thus, the E_S _value of HET or *F*_*ST *_in a particular gene represents the enrichment strength of the outliers of each measure. (Distributing from 0 to 1, a larger E_S _value indicates a higher outlier ratio of the corresponding measure in a gene.)

To estimate regional genetic diversity, we set up a 100 kb window and slid it with a 10 kb step throughout the human autosomes to obtain the averages of HET and *F*_*ST*_. This window scanning resulted in 267,069 and 266,650 regions (with 90 kb overlapping each other) in HapMap Phase II and Phase III data, respectively. Regional HET was first normalized within each chromosome and then the empirical distribution method described above was used to obtain window outliers of *F*_*ST *_and normalized HET. Adjacent window outliers were merged to regions and the qualification of each region was determined by a bootstrap test. Briefly, we picked 1,000 regions randomly with the similar length to the testing region, and then calculated the HET values of these picked regions plus the testing region. The percentile values of these 1,001 HET values were defined as *P*_*boot*_, and only those with *P*_*boot *_< 0.01 were accepted as low HET regions.

Consequently, 1,606 low HET regions were obtained from Phase II Build36 data, including 434 in ASN, 576 in CEU, and 596 in YRI. In addition, merged window outliers of *F*_*ST *_identified 660 highly differentiated regions among three groups. Moreover, 10,138 low HET regions were found from Phase III data, including 807 in CHB, 802 in CHD, 803 in JPT, 995 in CEU, 1,000 in TSI, 881 YRI, 965 in ASW, 916 in LWK, 962 in MKK, 1,023 in GIH, 984 in MEX. Additionally, 464 regions show strong differentiation among four geographical groups.

### Datasets containing evolutionary signals

**Figure 2:**
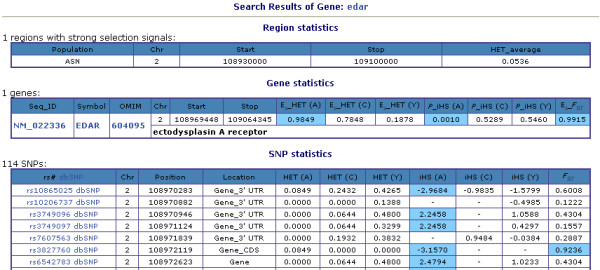
**Using EDAR to demonstrate the output of a data query**. After submitting EDAR as the search term, all hit data are provided in three tables. The regional statistics indicate whether the query is localized in a low HET region in a certain population or in a highly differentiated region among populations. The gene table with the sequence accession ID, gene symbol, and OMIM ID provides E_S _values of HET and *P*-value of iHS of each HapMap geographical group, as well as the E_S _value of *F*_*ST *_to show population differentiation. Finally, in addition to the rs# and its locations at both the chromosome and gene, SNPs of EDAR are displayed with individual HET and iHS of each population followed with *F*_*ST*_. The number above the third table indicates the total number of the SNPs identified in the query (only the first 7 were shown in the figure in this example). To illustrate selection related signals, the SNP outliers of iHS and *F*_*ST*_, as well as the extra E_S _values (>0.95) of HET and *F*_*ST*_, are highlighted in the tables.

To help users locate selection signals, SNP@Evolution describes the population genetic variations of chromosome regions, genes, and SNPs with measurements of *F*_*ST*_, HET and iHS (Fig. 2). The iHS obtained from Haplotter were first aligned with the corresponding HapMap SNPs. Of the 3,619,226 SNPs in HapMap Phase II Build36 that we computed with *F*_*ST *_and HET data, 2,663,137 SNPs also contain iHS information in Haplotter. Using the criteria of |iHS| ≥ 2 as defined in a previous study [25], 79,149, 86,272, and 100,624 SNP outliers of iHS in ASN, CEU, and YRI, respectively, were then obtained. In addition, 33,601 SNP outliers of high *F*_*ST *_from the empirical distribution method were found. As shown in Table 1, signals of large population differentiation are shown with the trend of increased strength from intergenic to genic regions (χ^2 ^test, *P *= 0.0000), suggesting that functional regions of the genome tend to be the targets of geographic selection. Additionally, the 5' UTR has the highest ratio of outliers. The outlier ratio in the 5' UTR is higher than other genic sections (χ^2 ^test, *P *= 0.0366, 0.0433, and 0.0728 for coding sequence, intron, and 3' UTR, respectively), implying the regulatory regions may play important roles in geographic differentiation.

**Table 1:** The distribution of *F*_*ST *_outliers across autosomes in data of HapMap Phase II Build36

		**Gene**						
				
		**5' UTR**	CDS*	**Intron**	**3' UTR**	**Total**	**Intergenic region**	**Total**
	**Outlier**	59	420	12,598	299	13,370	20,225	33,601
**SNP**								
	**All**	4,604	43,858	1,280,810	30,153	1,358,805	2,259,801	3,619,226

**Ratio (outlier/all)**		0.0128	0.0096	0.0098	0.0099	0.0098	0.0089	0.0093

* CDS, coding sequence.

As for all individual 24,011 genes listed with annotations, 21,859 genes with 5 or more genotyped SNPs in their genic regions are provided with E_S _values of HET and *F*_*ST*_, resulting in 1,094 genes of low HET which exceed the E_S _threshold of 0.95. In these 1,094 genes, ASN and CEU share 370, ASN and YRI share 147, CEU and YRI share 169, and all three groups share 81. Such a pattern--non-Africans sharing twice the number of genes containing enriched selection signals in comparison with that between non-African and African populations--suggests either geographical selection or founder effects by ancestors of ASN and CEU after their migration out of Africa. (Accordingly, we performed simulations based on both selection and neutral models. Very little founder effect was revealed (data not shown), suggesting that the selection events most likely resulted in these signals in genes. Our analysis results are consistent with recent reports which showed also geographic selections rather than founder effects as the major force of evolution in large genomic regions [29,30].) For the 535 genes of high *F*_*ST *_(E_S _threshold of 0.95), 369 were seen in at least one population which showing low HET (247 genes in ASN, 163 genes in CEU, and 42 in YRI respectively). In addition, *P*-values of iHS are included in 10,375 genes. For those with significant iHS (*P *< 0.05), 441, 527, and 433 genes are in ASN, CEU, and YRI, respectively, among which 32/441, 43/527, and 41/433 are also shown as low HET.

There are 1,389,498 genotyped SNPs in HapMap Phase III Build36, and among them, 351 outliers of *F*_*ST *_were found. E_S _values of HET and *F*_*ST *_in 21,099 qualified genic regions of all 11 populations are provided in our database. Finally, in our study we have found that for a fixed sample size and in regions with the same number of polymorphic loci, the regional HET is linear to Tajima's D [see Additional file 1]; therefore, we present HET data only in SNP@Evolution. Considering that iHS was derived from EHH and iHH, we have chosen iHS to be the measurement of haplotype diversity in our database.

## Utility and Discussion

There are two user interfaces in SNP@Evolution, one for data query and another for data visualization. In the data query interface, users submit one or more SNP rs# labels, gene symbols, gene sequence accession IDs, or specific chromosome regions. Results are displayed in three tables, as shown in the EDAR example in Fig. 2. Measurements--including the highly differentiated region among three geographical groups, E_S _values of HET, *F*_*ST*_, and *P*-value of iHS of the gene, as well as the HET, *F*_*ST*_, and iHS of the SNPs--indicate that the EDAR gene is under positive selection in Asians as previously reported [19,31]. Like all queries, these EDAR results can be downloaded in a table formatted file.

**Figure 3:**
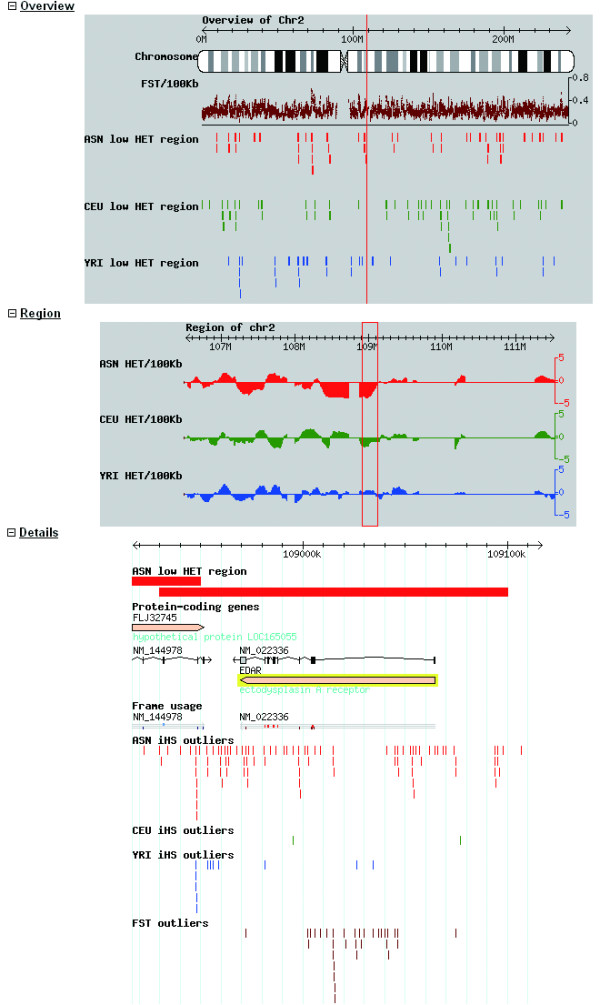
**Results of EDAR in the visualization interface**. Data are illustrated in three parts as Overview (chromosome scale), Region (area of searching target and flanking sequences), and Details (searching target). The Overview shows the *F*_*ST *_values of 100 kb sliding windows (maroon) and the low HET regions of merged window outliers in HapMap geographical group of ASN (red), CEU (green), and YRI (blue) along the ideogram of the chromosome. Region image displays the normalized HET values of 100 kb sliding windows that flanking the searching target in each population with the same color as illustrated in Overview. The basic line (zero) represents the average values of Normalized HET, therefore the comparatively high HET windows are shown above zero and low HET windows appear below the basic line. For the Details of the searching target, it provides (from the top to the bottom) the fine position in the chromosome, the low HET region (merged outliers of sliding windows) in the geographical group (red bar for ASN in EDAR gene), the genes (incarnadine) with the arrow pointing to the transcription direction, the transcripts and the frame usage of the genes (grey), the SNP outliers of iHS in each population as colored sticks, and at last the SNP outliers of *F*_*ST *_(maroon) to demonstrate if differentiation signals exist among three populations. For EDAR gene with a yellow label as the target gene, it is localized in a highly differentiated region among the geographical group and much more iHS outliers are in ASN than that in CEU and YRI groups. In addition, *F*_*ST *_outliers are also enriched in EDAR.

To better visualize the data, we implemented the Generic Model of Organism Database (GMOD) tool [32]. This interface uses similar input as described above. As shown in Fig. 3, each search result is shown as three parts as the Overview, the Region, and the Details. First, along the electronic G-stain of a given chromosome, *F*_*ST *_values of 100 kb sliding windows are depicted as scatter plots, and the low HET regions which were merged from adjacent window outliers are plotted as block diagrams (Fig. 3, top). Normalized HET values of 100 kb sliding windows are enlarged and illustrated as histograms (Fig. 3, middle). These figures allow one to directly observe the polymorphism and population differentiation patterns in chromosomes. In order to help locate positive selection signals throughout the genome, the SNP outliers of *F*_*ST *_and iHS are displayed as sticks in different colors along the searching regions, with additional gene annotation (Fig. 3, bottom). These features are provided with information of the three geographical groups shown in different colors.

Furthermore, results in the data query interface can be easily linked to the visualization interface to see the features under the background of the chromosome. To help users obtain additional information, we also provide the SNP links to HapMap database and NCBI dbSNP database and the gene links to Entrez Nucleotide and OMIM. To facilitate the operation, a page of 'Complete Guide' is included for detailed introduction and efficient use of SNP@Evolution. The terms used in measurements and population groups in this database are hyperlinked to the 'Abbreviations' page for brief reference.

Generally speaking, SNP@Evolution allows users to access all data through the 'Download' link. In these datasets, most of computations were conducted based on HapMap and related projects. As the data came from no more than a hundred of individuals in each HapMap population group, one shall also consider the sample size while making conclusions. The aim of SNP@Evolution is to provide genome wide signals of positive selection on human being, to generate fine scale traces of natural selection in enlarged samples is a long term goal in our research and computation.

## Conclusion

SNP@Evolution is a valuable and useful resource for finding and verifying signals of natural selection, and we will continue to update SNP@Evolution as research in positive selection progresses. At regions showing strong selection signals, we plan to add additional SNP information obtained from resequencing data from our work and from public datasets. Genotype data of new individuals from various sources will also be added to the database. By evaluating the effects of amino acid substitution with the method of Sorting Intolerant From Tolerant (SIFT) [33], we also plan to include protein functions at genic regions showing strong selection signals.

## Availability and requirements

SNP@Evolution is freely available at . A minimum screen resolution of 1,152 × 864 is recommended. Please send all questions, comments, and suggestions to chengf@big.ac.cn.

## Authors' contributions

The original data process and computation, webpage design, and manuscript preparation were undertaken by FC. WC made great contributions to the data integration, web organization and preparation of the manuscript. ER revised the manuscript and made great improvements regarding SNP@Evolution. LD analyzed the data and made important suggestions to the structure of the database. CZ directed the project and prepared the bulk of the manuscript. All the authors read and approved the final manuscript.

## Supplementary Material

Additional file 1 **Linear relationship between regional HET and Tajima's D**. The figure provided represents the statistical relationship between regional averaged HET and Tajima's D. The example is taken from Chromosome 22 of HapMap YRI group. Click here for file
