# Supplementary material for: SNP@Evolution: a hierarchical database of positive selection on the human genome
Source: BMC Evol Biol. 2009 Sep 5;9:221. doi: 10.1186/1471-2148-9-221 (PMC2755008; doi:10.1186/1471-2148-9-221)
Supplement: Additional file 1 — Linear relationship between regional HET and Tajima's D. The figure provided represents the statistical relationship between regional averaged HET and Tajima's D. The example is taken from Chromosome 22 of HapMap YRI group. [file 1471-2148-9-221-S1.doc]

**Supplement**


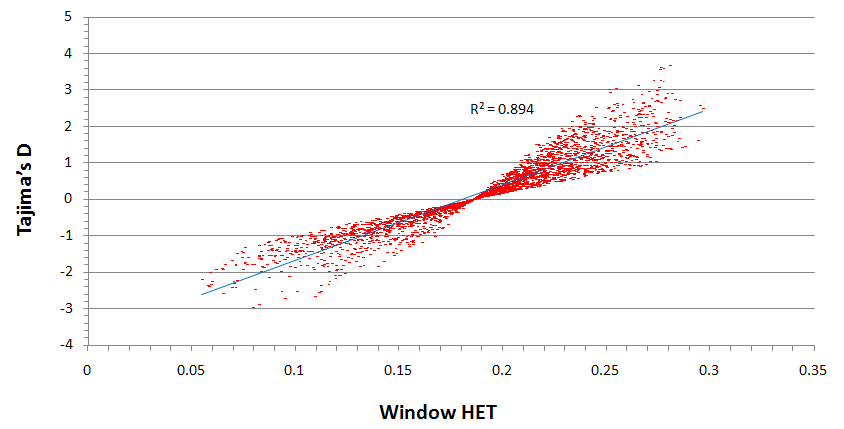


Sup. 1. In our analysis, we used a 100kb window with a step of 10kb on the human genome. When windows with more than 80 SNPs genotyped in the HapMap project are considered, Tajima’s D is linear to the regional averaged HET. As shown in the figure, Tajima’s D has a relationship with regional HET on chromosome 22 of HapMap YRI samples.
